# Low-Cost, High-Frequency, Data Acquisition System for Condition Monitoring of Rotating Machinery through Vibration Analysis-Case Study

**DOI:** 10.3390/s20123493

**Published:** 2020-06-20

**Authors:** César Ricardo Soto-Ocampo, José Manuel Mera, Juan David Cano-Moreno, José Luis Garcia-Bernardo

**Affiliations:** Railway Technology Research Center (Centro de Investigación en Tecnología Ferroviaria-CITEF), Mechanical Engineering Department, Universidad Politecnica de Madrid, 2 José Gutiérrez Abascal Street, 28006 Madrid, Spain; josemanuel.mera@upm.es (J.M.M.); juandavid.cano@upm.es (J.D.C.-M.); citef-jlgarcia@etsii.upm.es (J.L.G.-B.)

**Keywords:** data acquisition system, low cost, high sampling rate, Raspberry, vibration test bench, bearing diagnosis, envelope analysis

## Abstract

Data acquisition is a crucial stage in the execution of condition monitoring (CM) of rotating machinery, by means of vibration analysis. However, the major challenge in the execution of this technique lies in the features of the recording equipment (accuracy, resolution, sampling frequency and number of channels) and the cost they represent. The present work proposes a low-cost data acquisition system, based on Raspberry-Pi, with a high sampling frequency capacity in the recording of up to three channels. To demonstrate the effectiveness of the proposed data acquisition system, a case study is presented in which the vibrations registered in a bearing are analyzed for four degrees of failure.

## 1. Introduction

The progressive increase in industrial development has generated an obvious competition among businesses, who are seeking to maximize production and minimize operating costs. This gives rise to the need for ensuring the proper operation of mechanical equipment by means of condition monitoring (CM). This requires a high degree of sensing, that must be effective and depreciable in a short space of time, seeking to optimize the intervention timeline to allow the entire life cycle of each component to be exploited, and therefore to prevent unexpected downtimes and safety risks.

CM focuses on a three-stage development process, consisting of capturing records, data processing and information management [1,2]. However, the most serious drawback in implementing this technique lies in the recording equipment (precision, resolution, sampling frequency and the number of channels) and the cost involved [3]. These considerations mainly stem from the component to be analyzed and the measurement techniques used. One of the most widely used techniques in CM is “vibration analysis”, since vibration is regarded as one of the early onset unwanted phenomena [4,5].

Conversely, bearings are the most used components in rotating machinery and with the highest probability of failure, with only 10% reaching the end of their lifespan [6,7]. According to Nguyen et al. [8], bearings have caused up to 50% of the failures in rotating machinery. Nevertheless, these components require a high sampling frequency, since the presence of incipient failures generate waves that modulate at a high frequency. According to Bernal et al. [9], the preferred frequency for the diagnosis of bearings is 50 kHz, this limits the application of CM, since the equipment with these characteristics is quite costly.

It should be note that each CM phase plays a significant role. In order to maintain consistency between these phases, the ISO 13373 standard [10,11], provides a detailed guide of the parameters to consider in each phase of CM development. It also explains that some of the considerations may be employed in other equipment not included in the standards. Meaning that the environment characteristics must be personalized, such as speed, load, temperature, power and other characteristics that may affect vibration measurement [10].

In general terms, the collection systems used are selected among those available on the market. The cost of this equipment approved for registering vibrations is drastically high, over 4000€ for one channel collecting equipment, such as the ADASH 4900 VIBRIO or RH71, increasing its value for a larger number of channels. There is other lower cost Gani and Salami equipment [12].

Conversely, commercial registering equipment such as USB-231 or Labjack U6 to name a few examples, are considered low-cost (around 270€ and 300€ respectively). They are capable of acquiring data from more than one channel at a time, but with a unit maximum sampling frequency limit of 50 kHz, which means using one single channel for analyzing certain components such as bearings. Nevertheless, a lower sampling frequency can be used to record more components such as can be seen in [13]; however this involves sacrificing masked high frequency information.

In addition, there are several studies of prototypes of data acquisition systems [14,15,16,17], but most lack the data for reproducibility, the collection capacity does not meet the vibration requirements or they contain a small number of channels. Bosso et al. [14], present a multifunctional system prototype applied to railways, based on vibration and temperature collection; however, they do not test the efficiency of their algorithm relating to bearing vibration. Moreno et al. [15] display a multi-sensor data acquisition system, capable of registering high frequencies. Nevertheless, they require a computer connection to control the capturing of records, a feature that limits implementation in equipment which is difficult to access that operates in movement. Vidal and Pindado [16] present a 5-channel Arduino-based data acquisition system, however the sampling frequency is estimated at 500 Hz.

In order to deal with this situation, this work proposes a low-cost data acquisition system model based on Raspberry-Pi with a high sampling frequency when registering through each one of its three channels. This model effectively meets the necessary conditions for the analysis of bearing vibrations, which are components used in over 90% of rotating machinery that have a high fault probability [6]. In addition, a practical case is presented using test bench modelling and the record captures of recordings of bearing vibrations simulating faults in rolling elements with four severity levels, in order to demonstrate the performance of the proposed system. Ultimately, the information of the vibration signals requires analysis by means of processing, for which an interface has been developed for processing the recordings in Matlab^®^, using the envelope analysis technique. To minimize the impact on cost, it is planned to program this analysis in an open source environment, such as C++ or Python.

## 2. Materials and Methods

### 2.1. Data Acquisition System Design

The design of the data acquisition equipment has been carried out according to the bearing vibrations analysis requirements, making it essential for data to be acquired through several channels with a sampling frequency of up to 50 kHz for each one and compact enough to be easily placed in components that are difficult to access. In order to meet these requirements a low-cost small-sized Raspberry PI 3 B+ computer board has been selected. This unit has an independent operating system, supports several programming languages, with remote communication capacity, operational control and data storage.

The operation of the Raspberry Pi is not capable of acquiring signals from analogue sensors, since it does not include the analogue to digital conversion hardware, therefore requiring a prior stage using an external converter.

#### 2.1.1. Raspberry Pi Specifications

The Raspberry Pi (RPi) is a computer with features that meet the requirements outlined. In has an integrated Broadcom BCM2837B0 chip containing a Cortex-A53 64-bit processor, that operates at 1.4 GHz clock speed, 1 GB memory, a 2.4 GHz and 5 GHz dual band wireless connection and Ethernet Gigabit over USB 2.0 that supports up to 300 Mbps and a power supply of 3.3 and 5 V.

It has a series of general purpose input/output (GPIO) pins, that represent the communication interface between the RPi and the environment. The main feature of the GPIO is that it can designate a pin as a data input or output in the software, as required. For this purpose, it incorporates communication interfaces for digital sensors type I2C, SPI and UART. In the development of this project, registration tests were carried out using I2C and SPI converters. Due to the data transfer speed being one of the main requirements, this project uses an SPI communication protocol between the Raspberry Pi and the A/D converter, since it has two data transmission lines [18].

The SPI communication interface devices have a synchronous protocol that operated in full duplex mode, to receive and transmit information through a single cable, dividing the cable into several channels to do so. Communication between both is carried out by means of a bus with 4 logic signals (MOSI, MISO, SCK, SS). The MOSI and MISO ports carry the signals in both directions, the SCK pin is the clock signal that synchronizes the data reception, and finally, the SS pin activates the corresponding slave.

#### 2.1.2. Analogue/Digital Converter (ADC)

The data acquisition and processing devices operate in a digital environment by means of an 8 bit binary value. For this purpose, the analogue variables provided by the sensors must be converted into binary equivalents. One of the main characteristics to bear in mind when selecting an ADC is the relationship between the sampling frequency and the resolution, given that it provides the capacity to obtain the positioning of the frequency and its corresponding amplitude with greater precision and sensitivity [11]. Furthermore, the communication protocol is added to these requirements, given that the ADC must allow an SPI communication in order to transfer data to the RPi.

Using these considerations, it is proposed to implement an ADC based on successive approximation register (SAR), since it is ideal in data acquisition processes, where accuracy is relevant. There are some ADC models based on this architecture with similar characteristics, such as ADS8326, AD7694ARMZ and LTC1864ACS8#PBF. After tests developed, the best performance was observed in the ADS8326, additionally its low power consumption allowed the development of a more compact and portable device. Its main features include a resolution of 16 bits, obtaining a dynamic range of 90 dB, a voltage supplement (V_DD_) of between 2.7 and 5.5 V, a sampling frequency (F_sample_) of 250 kHz, a synchronous serial interface compatible with SPI, excellent linearity and very low noise and distortion. It also incorporates a differential input and a reference voltage (V_ref_) may be configured from 0.1 V to V_DD_. This V_ref_ is undertaken by NPC1541, which provides a low power voltage and high precision, set at 4.096 V; this is used by SAR logic to compare and determine the binary correspondence of the analog input value.

**Figure 1:**
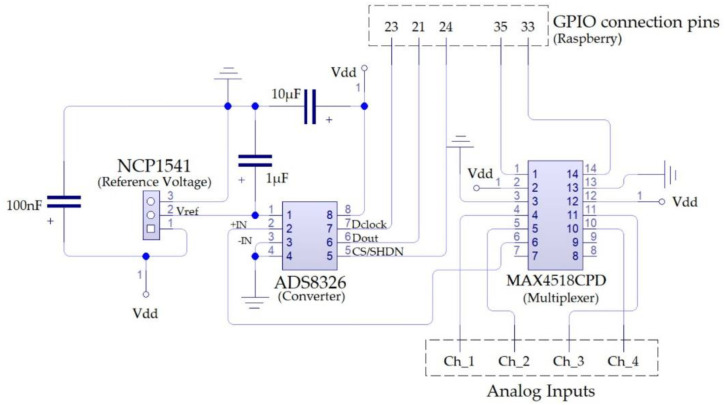
Analog to digital converter (ADC) electrical wiring diagram.

The ADC performs the function of quantifying and coding the amplitude measurements transmitted from the sensor. This quantification requires that the samples taken be retained long enough to evaluate their level. Therefore, a sampling and retention circuit is required. Said function is undertaken by the MAX4518CPD analogue multiplexer, made up of four compatible channels with CMOS logic and a fast switching speed (transition time less than 250 ns), which enables several nodes to communicate at the same time with a shared transmission medium. The Figure 1 shows the connection circuit diagram used in this project.

#### 2.1.3. Sensors

The design of the data acquisition system must be versatile regarding the number of channels recorded. As a result, the hardware and software must be capable of adapting to the specified configuration as required. According to ISO 13373-1 [10], the transducer used depends upon the component to be analyzed, recommending accelerometers to quantify the vibration rate in bearings, as they may display masked faults in high frequency.

**Figure 2:**
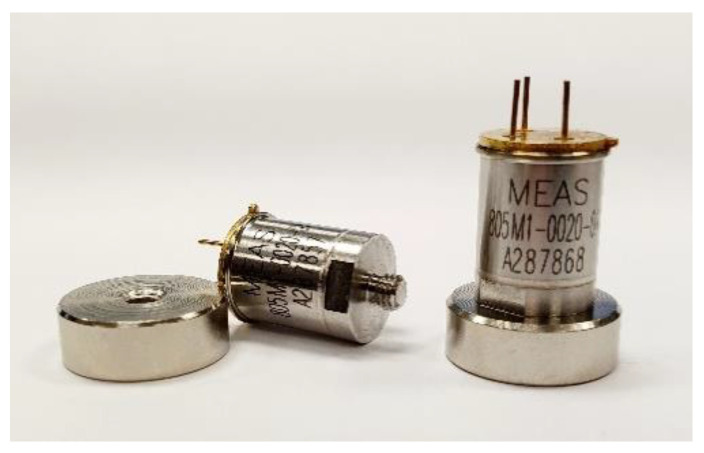
805M1-0020 accelerometers.

This acquisition system uses a low-cost 805M1 accelerometer model and a threaded connection assembly. It has a dynamic range of ±20 g, a sensitivity of 100 mV/g and a flat frequency response of 0.4 Hz to 10 kHz (±3 dB). This transducer incorporates a stable piezoceramic crystal, with an excitation voltage of 3 to 5.5 V and an operating temperature between −40 to +100 °C. The Figure 2 shows the accelerometer used in this project.

#### 2.1.4. Programming the Raspberry Software

**Figure 3:**
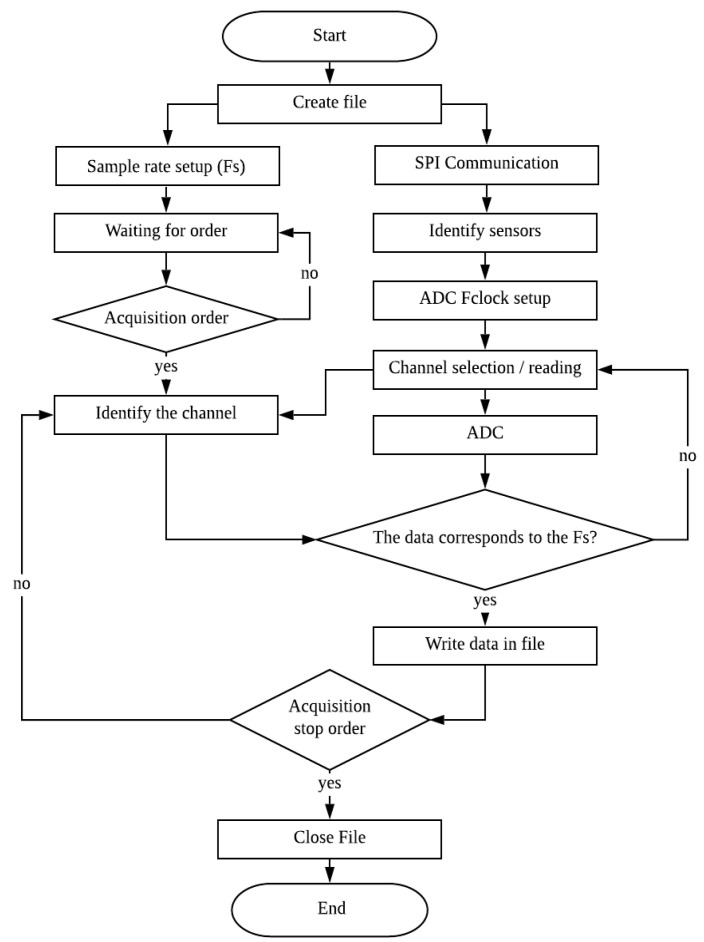
The data acquisition system operational flow chart.

The hardware designed requires a software for control. It must be intuitive and user friendly to configure, in terms of the number of channels and sampling frequency to be used. The code was developed in C++, using a BCM 2835 library on the Broadcom chip for GPIO pins access and control, the flow chart is shown in Figure 3.

Once the register interface has been initiated, a “.txt” file is created and saved in the RPi’s SD memory. In addition, a multithread system is established, where the data corresponding to the requested sampling frequency is stored in a queue, and an independent writing thread system, where the data of the storage queue is dumped to be written in the “.txt” file. Afterwards, two flow lines are established. One of these recognizes the SPI communication protocol defined in the executable, it configures the number of sensors and sets the converter clock’s maximum working frequency ($F_{clock}$). Said frequency is estimated according to ADC specifications, $F_{clock}=24\cdot F_{\mathrm{Sample}}$. Subsequently, a data channel, reading and conversion selection loop is initiated.

The remaining line requests the recording configuration, requesting the sampling or writing frequency in this case, enabling the time interval to be estimated in which the data are written. Once the writing order has been initiated an identification loop of the prevalent reading channel and correspondence comparison of the data is initiated with the established writing time interval. In the event it does not match, the data is discarded until the condition is met, this is done independently for each channel. Conversely, if it does match, the value is stored in the multithread, for later dumping into the write thread written in the “.txt” file; and the process is repeated until the detain writing order is executed, closing the file and concluding the program.

In order to write the data of each channel a time vector and an amplitude vector has been created. The process starts when the desired sample rate is set, and the data capture is started. The time vector is provided by software, using a high-resolution timer (time.h library). Using the function “clock_gettime” a real-time clock is accessed through the CLOCK_MONOTONIC function, with 8-bit precision. The time vector starts at zero and is always increasing based on real time. As the timer rate is higher than the sampling rate, when the time provided by the function above is a multiple of the required sampling frequency, the time vector is accessed and the value saved for each channel. Since the N channels are connected through a multiplexer up to 1 *×* 4, the time of each channel varies slightly.

As defined in the software structure, data writing is not achieved at a constant frequency, it therefore requires data processing by means of subsequent resampling. In addition, the recordings do not specify the characteristics of the accelerometers (sensitivity and offset), the data obtained being in volts. Therefore, they must be regarded in the subsequent processing of recordings, in order to obtain an appropriate definition of the magnitude of the signal. These observations will be included when creating a future code.

#### 2.1.5. Connecting the Data Acquisition System

**Figure 4:**
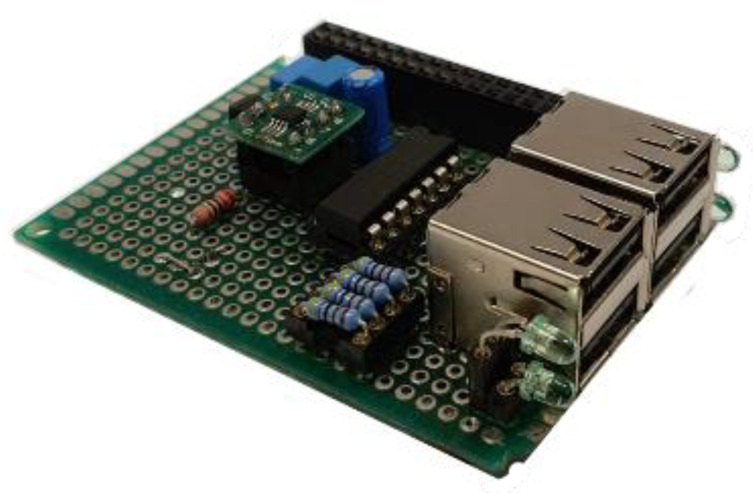
Design of the conditioning and data conversion board.

One of the data acquisition system’s priorities is managing to achieve a compact size, enabling an easy integration of the system into hard-to-reach sites. Requiring all the components to be integrated into a PCB board. The board must allow an easy and quick connection to the RPi, in order to enable this a GPIO 40 pin adaptor has been used, only connecting the pins configured with the SPI protocol. This PCB board has four USB version 2.0 ports to link the accelerometers, as is shown in the Figure 4.

**Figure 5:**
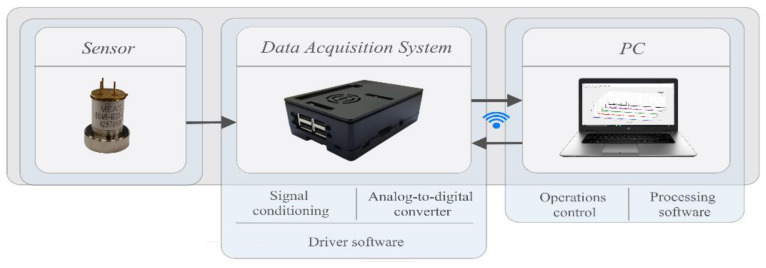
Components of the data recording system.

In addition, the recording capture management is carried out by remote control connection. This property eliminates communication by cable with the recording equipment, enabling remote recording capture management and providing greater accessibility to mechanisms that operate at a distance. These features enable usage on a larger number of mechanical units. Figure 5 shows the components of the data acquisition system.

### 2.2. Bearing Test Bench

**Figure 6:**
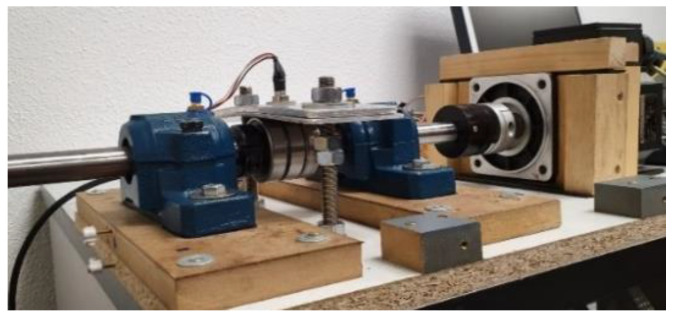
Bearing test bench.

The test bench is prepared for validating the recording equipment, by means of capturing the recordings of faults arising in the bearings. In addition, the equipment must be able to function under different operating conditions in terms of regime and load, since these are considered to be the main parameters influencing the vibration rate [19]. The layout of the proposed test bench is shown in the Figure 6.

#### 2.2.1. Traction Unit

Traction is generated by a series BL 110 synchronous electric servomotor, enabling changes in revolutions with a high dynamic response. The control is carried out by a CD1-a amplifier, with a PMW module to regulate torque and speed, and a “resolver” transmitter, for feedback and stabilizing the parameters.

#### 2.2.2. Bearing under Study

When selecting the test bearing, the following have been considered: the shaft dimensions (20 mm), accessibility to the bearing and ease of assembly and disassembly. Furthermore, the results of this project seek to be extrapolated in future research, to railway grease boxes, hence the need for using bearings with geometric and constructive characteristics similar to the real ones. Manufacturers such as SKF, FAG or NSK to name just a few, offer a wide range of bearings for grease boxes, with double-row roller elements and configurations from cylindrical to tapered rollers, depending on their application.

**Figure 7:**
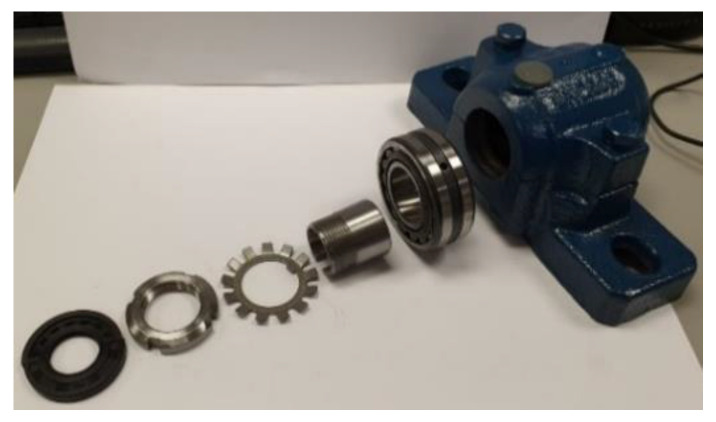
Dismantling of the test set.

Therefore, bearing in mind the restrictions and requirements mentioned previously, this project used an SN-505 split casing, that will act as the grease box. The casing will contain a 22205E1KC3 double-row spherical roller bearing and TSNG-505 blocks enabling the sealing of the casing and the shaft. The bearing is fastened to the shaft using a H-305 sleeve, as shown in the Figure 7.

##### Bearing Vibration Mode

A bearing is a mechanism that, in operation has its own vibrating signal, caused by the contact between the components it is made up of and interpreted through its fundamental frequencies. This signal is defined by the kinematics of the rolling elements and the Hertzian or elastic contact of these with the rolling tracks. Establishing a relationship between the geometry of the components and the energy developed when the rolling elements pass over the load area [20].

**Figure 8:**
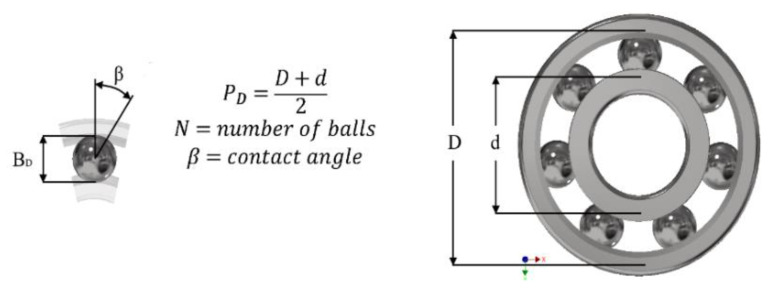
Calculation of the fundamental frequencies of the bearing. In accordance with the geometric characteristics of the bearing used, a table of the frequencies of each component, for different rotating regimes is shown below. These frequencies have been taken from the official Schaeffler webpage.

Therefore, the presence of irregularities generate changes in the contact forces of the bearing components, resulting in the generation of short duration pulses that excite the natural frequencies of the entire structure, between the bearing and the response accelerometer, modulated in high frequency ranges [7,20,21,22]. Figure 8 indicates the geometric characteristics used in the calculation of the frequencies and Table 1 details the frequencies at the studied speeds. The equations to calculate the frequency of the outer ring (BPFO), inner ring (BPFI), rolling elements (BSF), and the cage (FTF) are shown below.

(1) $$\begin{array}{cc} BPFO=RPM\cdot \frac{N}{2}\cdot \left(1-\frac{B_{D}}{P_{D}}\cos\beta \right)\; & BPFI=RPM\cdot \frac{N}{2}\cdot \left(1+\frac{B_{D}}{P_{D}}\cos\beta \right)\\ BSF=RPM\cdot \frac{P_{D}}{B_{D}}\cdot \left[1-{\left(\frac{B_{D}}{P_{D}}\cos\beta \right)}^{2}\right]\; & FTF=RPM\cdot \frac{1}{2}\cdot \left[1-\left(\frac{B_{D}}{P_{D}}\cos\beta \right)\right] \end{array}$$

#### 2.2.3. Load

**Figure 9:**
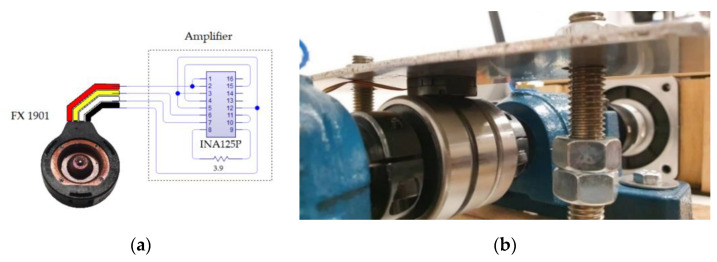
Compression load cell: (**a**) electrical diagram and (**b**) positioning in the test bench.

According to ISO standard 13373 [10], load is one of the most influential factors in the vibration rate of rotating machinery, since the vibration rates vary significantly under normal load and no load operations. Therefore, a threaded tightening tower has been used, achieving a load of 1.4 kN for a tightening torque of 7.4 Nm. Figure 9 shows the tightening tower used in this study.

An FX1901 compression load cell was used for the calculation, analyzing the ratio between the voltage, weight and the tightening torque. Due to the linearity observed in the two ratios up to 200 lbf (the capacity of the compression load cell), the tightening torque required for a weight of 150 kg was calculated by extrapolation. This compression load cell uses an INA125P amplifier to condition the output voltage (0–5 V) to the load capacity (0–200 lbf). On this load transmission three SKF 6304-2R spherical bearings were used on the shaft, the operational frequencies of which were taken from the official SKF webpage and are shown in the Table 2.

### 2.3. Sensing

This section is systematized according to ISO standard 10816-7 [19]. Considering the “test bench” is structured horizontally and the bearings are the analysis components of interest, the orientation of the accelerometer will be perpendicular to the shaft and located on the bearing casings, as close as possible to its center. This study uses one accelerometer for each bearing, and the recordings are performed in the three accelerometers at the same time. However, although the data recordings are executed together, this study focusses on the analysis of a bearing, connected to the accelerometer “Ac_1”. The monitoring of the three bearings is carried out in order to ensure that the discrete frequencies observed correspond to the bearing under study and for acquiring more information, related to two different bearings, data that in future work will enable the generation of the diagnostic algorithms.

**Figure 10:**
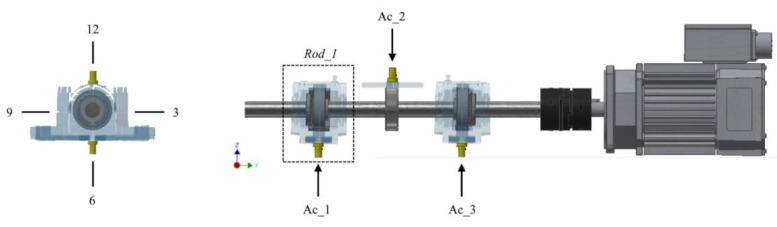
Positioning and orientation of accelerometers.

On the other hand, it is important to obtain the greatest sensitivity to changes in the vibratory behavior. Therefore, the areas of the maximum dynamic forces of the bearings being used are identified, establishing an orientation of the accelerometer of the bearing casings at 6 o’clock (lower part), and at 12 o’clock (upper part) for the load bearing, as suggested by Smith and Randall [23]. The orientation of the accelerometers is shown in Figure 10.

In order to ensure maximum rigidity between the accelerometer and its respective positions an adhesive assembly has been used, to ensure the transducer signal does not undergo major distortions due to the resonance frequency of the type of assembly [24]. It should be emphasized that, due to the constructive characteristics of the transducer, where the point of contact is threaded (Figure 2), a magnetic coupling has been used to ensure the perpendicularity of the accelerometer with the contact surface.

### 2.4. Design of Experiment (DoE)

**Figure 11:**
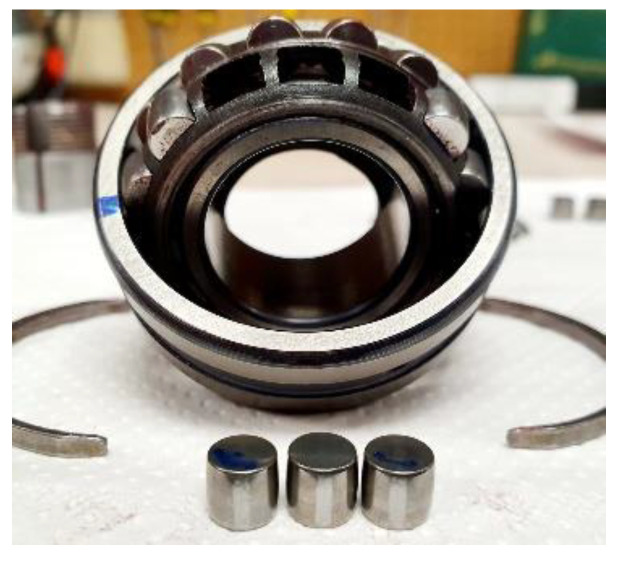
Diagram of the position of the REs with induced faults.

The DoE has been devised to validate the high frequency sampling capacity of the recording equipment, as well as its precision and sensitivity. Using a test based on the induction of faults in three rolling elements (RE) of a bearing, designed as “Rod_1”; since according to [23] in the study, this component is highlighted as being the most difficult to diagnose. It should be emphasized that, as the bearing is made up of two rows of REs, the three REs are positioned on one of these, as shown in the Figure 11.

This test is based on a “Factorial 5 × 3” design, to analyze the effect of the five RE fault levels (F0, F1, F2, F3, F4) and the three operating regimes (200, 350, 500 rpm), on the bearing vibration mode. Each process was run three times, therefore giving a total of 45 recordings. The repetitions are carried out after the fault of each rotating regime has been evaluated, in order to ensure that minor differences due to uncontrollable variables are distributed homogenously throughout all the recordings. The factors and levels considered in the test are shown in Table 3.

**Figure 12:**
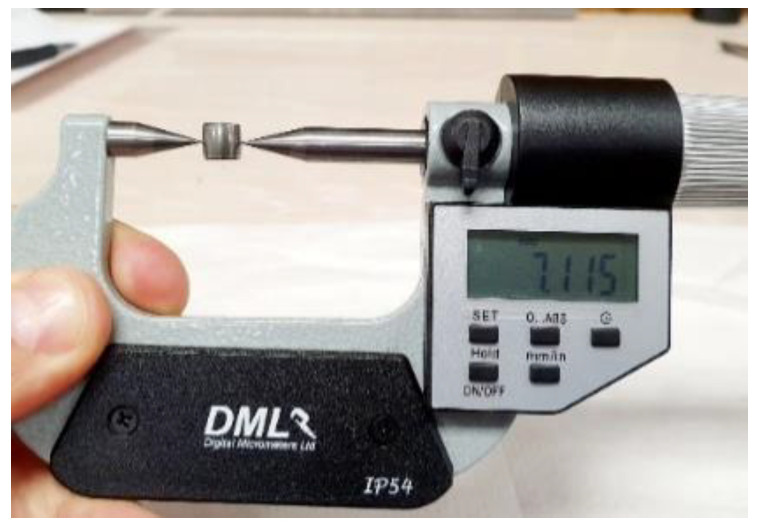
Quantification of the degradation generated in rolling elements.

The depth of the fault generated in the RE is expressed as an absolute magnitude of the difference between the normal diameter and the induced fault. The development of the fault in the REs is generated by milling the surface of each component, and quantified using a DML IP54, with a 1µm minimum unit of measurement, as shown in Figure 12.

One of the main requirements in analyzing vibrations in bearings is the high sampling frequency [9], a feature that validates the recording equipment proposed. Therefore, these recordings were taken at the equipment’s maximum sampling frequency, of 45 kHz to be used in 3 channels; the equipment’s recording capacity is detailed in Section 3.1. In the subsequent data processing, the same 40 kHz are resampled in order to ensure a constant sampling frequency throughout the entire recording.

Table 4 provides details of the conditions of the medium (uncontrolled variables) in the development of the experiment, since these are very important variables and may influence the results of the experiment [25].

Considering the DoE is made up of three replications for each treatment, the one that showed the most representative value in the experiment was evaluated. The identification of the experimental unit or interest replication, will enable its treatment and representation in the analysis of the fault evolution. For this an analysis between replications, the correlation (Cor), covariance (Cov) and coherence (Coh) of each treatment was determined within the frequency domain by means of FFT. A list of the relationship developed for each treatment is shown below, comparing their replications.

(2) $$\left[\begin{array}{ccc} Cor_{1,2} & Cor_{1,3} & Cor_{2,3}\\ Cov_{1,2} & Cov_{1,3} & Cov_{2,3}\\ Coh_{1,2} & Coh_{1,3} & Coh_{2,3} \end{array}\right]$$

Subsequently, in order to determine the most representative replication of each treatment, the “geometric mean” (µR,i) was determined between each ratio estimator contained in the replication under analysis and between the three estimators analyzed, selecting the one with the highest value, which indicates a lower data dispersion and ensuring the absence of atypical values in the recordings.

(3) $$\left\{ \begin{array}{l} \mu_{R1}=\sqrt[{3}]{\sqrt{Cor_{1,2}\cdot Cor_{1,3}}\cdot \sqrt{Cov_{1,2}\cdot Cov_{1,3}}\cdot \sqrt{Coh_{1,2}\cdot Coh_{1,3}}}\\ \mu_{R2}=\sqrt[{3}]{\sqrt{Cor_{2,1}\cdot Cor_{2,3}}\cdot \sqrt{Cov_{2,1}\cdot Cov_{2,3}}\cdot \sqrt{Coh_{2,1}\cdot Coh_{2,3}}}\\ \mu_{R3}=\sqrt[{3}]{\sqrt{Cor_{3,1}\cdot Cor_{3,2}}\cdot \sqrt{Cov_{3,1}\cdot Cov_{3,2}}\cdot \sqrt{Coh_{3,1}\cdot Coh_{3,2}}} \end{array}\right.$$

### 2.5. Data Processing

As in the recording phase, “data processing” is another critical phase for the success of the diagnostic process using condition monitoring [26]. This factor is considered in [11] as a set of activities used for participating in the signals acquired, developing the filtering of noise or other undesired signals, formatting signals of interest and applying various available representation methods, in order to facilitate the identification of the characteristics of the mechanism under study by the analyst.

Gupta and Pradhan [20], suggest that the data processing technique must be considered in accordance with the analysis component. Therefore, considering the bearing’s oscillating fundamentals, this study uses the “envelope analysis”, given that it mainly seeks to detect resonance areas excited or modulated in amplitude by periodic impact forces, whose repetition frequency is an indicator of where the defect is located and its amplitude, a measurement characterizing the status of the component [27]. This process involves the use of a sequence of operations with the vibration signal, that initiates by eliminating the low frequency components associated to other conditions of the rotating equipment, such as imbalance and misalignment [28]. The signal’s envelope is then determined, obtained in this study by using the Hilbert transform.

The removal of interference components is executed by filtering the signal, making it necessary to determine the band of frequencies to analyze, in order to obtain the greatest amount of information on the status of the bearings. McInerny and Dai [29], describe that most of the diagnostic equipment enable the analysis of signals in bands of 1–2.5, 2.5–5, 5–10, 10–20 and 20–40 kHz. As an example, the diagnostic and data collector unit “Adash 4900 Vibrio Ex”, uses 3 bands (0.5–1.5, 1.5–5 and 5–16 kHz) for detecting faults at high frequency. In this work, the selection of these bands is described in Section 3.2.

## 3. Results and Discussion

In order to investigate the performance of the recording equipment proposed for analyzing mechanical vibrations, this section presents the results obtained by means of recorded data processing. This database is available, the section “Supplementary Materials” is the access link. Similarly, a brief description of the recording equipment is presented and the fundamentals considered in the selection of the band-pass filter. Subsequently, the results defined in Table 3 of the fault diagnostics in rolling elements are discussed. 

### 3.1. Validation of the Recording Equipment

This section details the tests performed to validate the recording equipment regarding its performance and sampling capacity through each channel. The equipment’s performance evaluation consisted of the capture of known frequency waves (gradually increasing their frequency) generated by an oscilloscope, observing a high precision in their identification.

In order to learn about the equipment’s recording capacity per channel, a quantitative analysis is performed to determine the maximum sampling frequency when using a certain number of channels. This confirms the design of the equipment proposed, for its high frequency data acquisition capacity. This study is based upon data acquisition at a frequency of 110 kHz, for different numbers of channels, and a length of 3 min. The results show that, as the number of channels used increases, the maximum sampling frequency is reduced. Table 5 with the analyzed results is shown below, detailing the maximum average sampling capacity per number of channels employed and the standard deviation by which the sampling frequency fluctuates.

As can be observed, the recording equipment proposed can achieve a sampling frequency of over 100 kHz using one channel. This represents an advantage when compared to other recording equipment, such as the USB-231 or Labjack U6; the maximum sampling frequencies of which for one channel are less than half of those achieved in this study. Another representative advantage is related to its cost; which, depending upon the supplier of the main components, can be obtained from 70€ (without considering the sensors); an estimated value of three times less, when compared respectively with the equipment mentioned beforehand.

### 3.2. Determining the Analysis Band

The estimate of the analysis band is based on the study of discrete frequency components proposed in [23], where by using the “power spectral density” (PSD) technique, it evaluated the band frequencies in which the discrete components tend to increase their amplitude. This analysis was performed on the experimental units of each treatment, observing similarities in the bands proposed, even when increasing the rotating regime. As an example, the following shows a representation of recording 15, associated to the first fault level in rolling elements. This figure, suggests frequency bands of (0.2–1.8 × 10^3^; 1.8–6.1 × 10^3^; 6.1–9.2 × 10^3^ and 9.2–12.8 × 10^3^ Hz) for the filtering prior to the treatment of signals, where it has been observed that the discrete components tend to increase energy.

**Figure 13:**
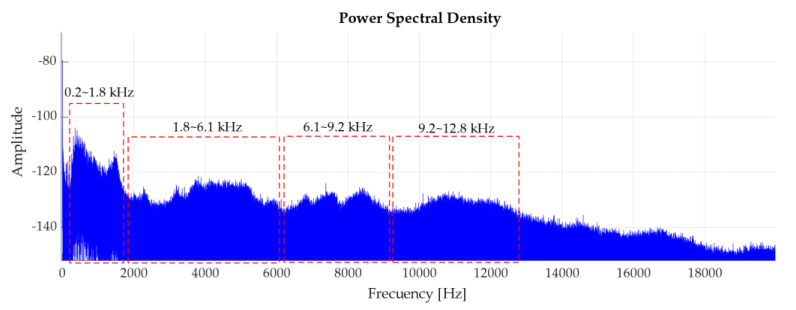
Power spectral density of the record 15 (40 kHz, rolling element failure, fail level 1, regime the 500 rpm). Proposed bands for envelope analysis.

The following figure represents the envelope analysis of the bands suggested in Figure 13, where the higher areas of signal energy were observed. In cases a and b, the increase in amplitude of the frequencies close to characteristic BSF frequencies and their respective harmonics is evident; unlike cases c and d, where the frequency amplitudes of interest are distorted by the background noise. Although the first two cases are the most relevant, case a shows a large presence of FTF, rotation frequency, respective harmonics, and frequencies not related to the fault characteristic, that show less influence in case b. Conversely, the number of harmonics related to the fault is more noticeable in case b.

**Figure 14:**
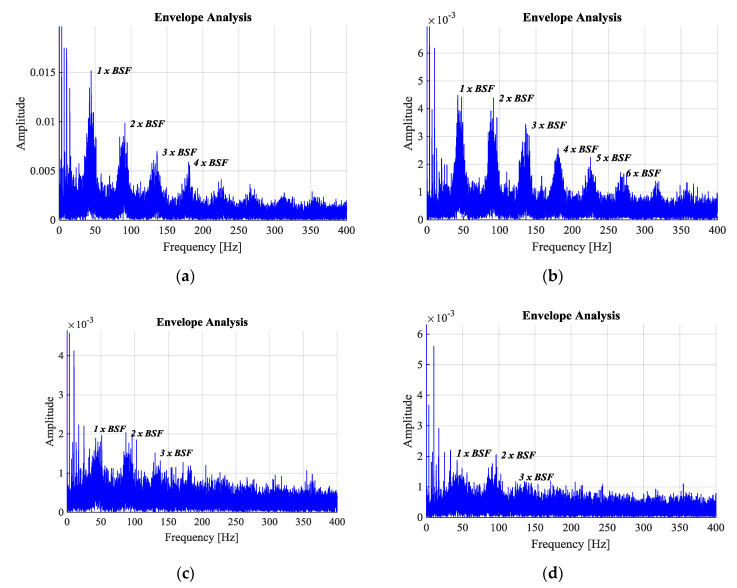
Envelope analysis of recording 15, using bands: (**a**) a band of 0.2–1.8 × 10^3^ Hz, (**b**) a band of 1.8–6.1 × 10^3^ Hz, (**c**) a band of 6.1–9.2 × 10^3^ Hz, (**d**) a band of 9.2–12.8 × 10^3^ Hz.

According to Carter [30], the amplitude modulation generated by defects in the bearing will be quickly attenuated, as the analysis frequency increases. This can be seen in Figure 14, where, as the analysis band increases the amplitudes of the frequencies of interest decrease. However, observing the background noise and the easy identification of the characteristic fault frequency, this study uses the second band suggested, between 1.8 to 6.1 × 10^3^ Hz; where more precision was observed in identifying the frequency and harmonics of interest.

### 3.3. Detection of Faults in “Rolling Elements”

**Figure 15:**
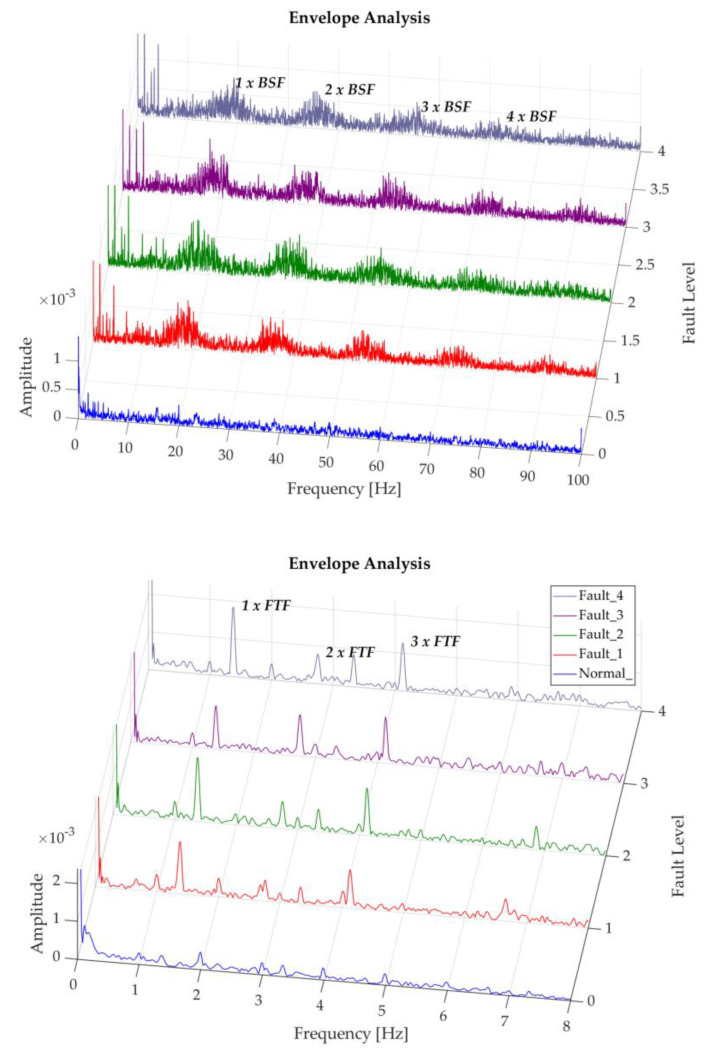
Representation of recordings of rolling element degradation at 200 rpm.

The following shows the degradation of the rolling elements in this study for a regime of 200 rpm, in which it is difficult to identify the fundamental frequency BSF or its harmonics. Nevertheless, an increase in trend of the frequencies close to BSF is observed and its corresponding harmonics, as the severity of the fault grows, as shown in Figure 15. Smith and Randall [23], define the classical envelope spectrum vibration mode for rolling elements as a representation of two indications, the first of these manifested as harmonics of BSF with side bands modulated at FTF frequency, and the second corresponding to low amplitude FTF harmonics. However, very few bearings with these faults present these symptoms, making them difficult to diagnose. Conversely, the following figure shows a clear presence of the second indication in the spectrum obtained, with representative amplitudes; which according to Graney and Starry [6], are an indicator that the bearing is reaching the end of its service life. This characteristic shows that the magnitude of damage caused in the rolling elements of this study is drastic. In this case a change in the vibration spectrum can be seen between the normal recording and the recordings with induced faults, maintaining a similar behavior between the induced fault levels.

**Figure 16:**
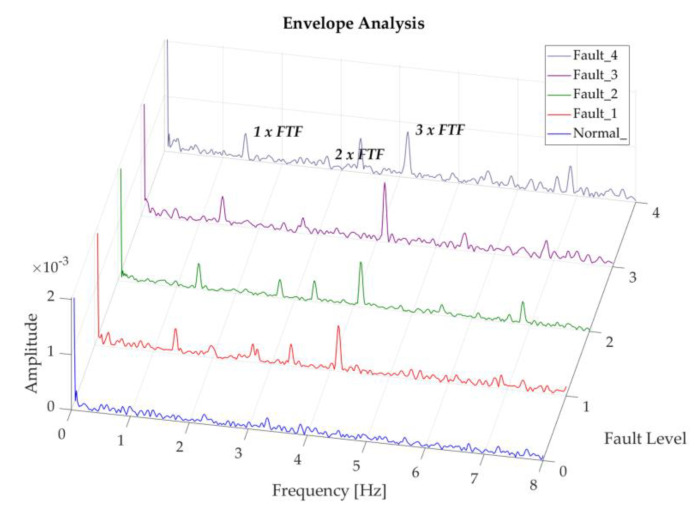
Representation of recordings of rolling element degradation at 200 rpm, analysis band of 9.8–12.8 × 10^3^ Hz.

The three first FTF harmonics can be observed in the results. This number of harmonics coincides with the number of damaged rolling elements in this test, being more noticeable in the envelope analysis performed in the 9.2–12.8 × 10^3^ Hz band, where there is a greater representation of 3 × FTF for this case, as can be seen in Figure 16. As was mentioned beforehand, this evidence is an indicator that the bearing is getting close to collapsing.

**Figure 17:**
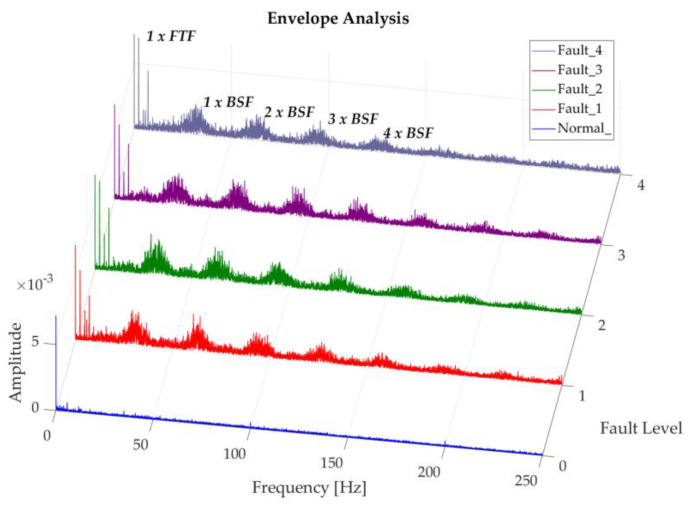
Representation of the Envelope of recordings of the rolling element degradation at 350 rpm, in a band of 1.8 × 10^3^ to 6.1 × 10^3^ Hz.

Figure 17 shows the representation of the degradation of rolling elements at 350 rpm; in which a behavior similar to the one above can be observed. This spectrum also shows an impulsive content in the side bands of the BSF harmonics, which grows as the fault severity increases. This criterion is taken into consideration by Smith and Randall [23], attributing two possible causes as the answer, the first as a result of the amplitude modulation in response to random impulses, that are not related to the specified fault; the second is due to the presence of extraneous loose debris which cause occasional pulses.

Since the bearing was dismantled, cleaned and lubricated in each fault induction, ruling out the possibility of impurities in the lubricant and the behavior of the bands is attributed to, the amplitude modulation of successive impulses of a random nature. This impulsive behavior generated by faults in RE has also been observed by Amini et al. [31], in his analysis of a severely damaged railway grease box bearing.

**Figure 18:**
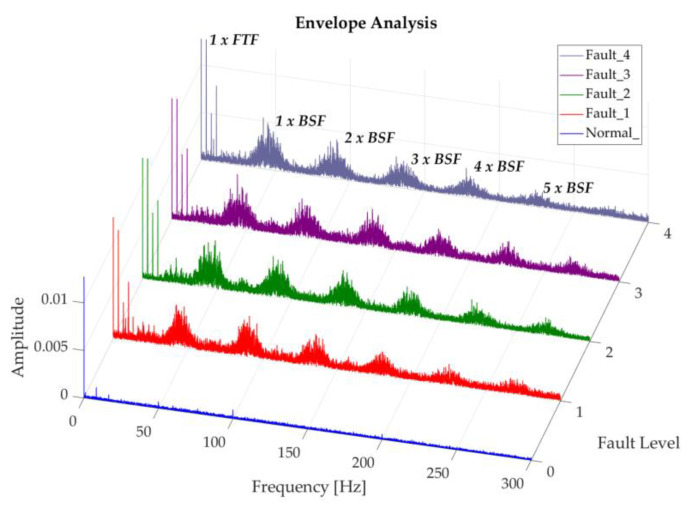
Representation of recordings of rolling element degradation at 500 rpm.

Figure 18 represents the degradation spectrum of rolling elements at 500 rpm, the behavior of which is similar to the previous ones and where the influence of the rotating speed is noticeable in the 1 × BSF amplitude, which is more than five times and almost double of the degradation at 200 and 350 rpm respectively, when comparing the more severe fault cases in each operating regime.

**Figure 19:**
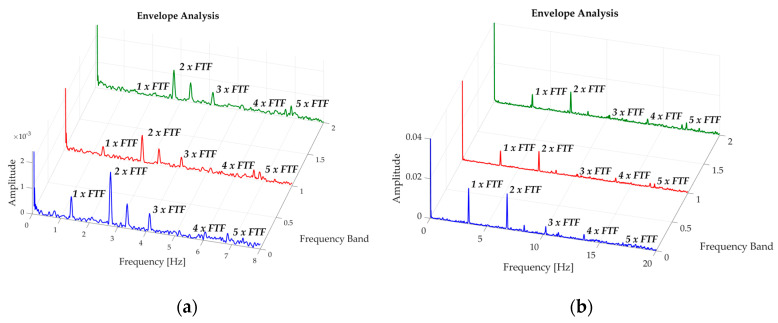
Envelope Analysis for a bearing with 5 damaged REs, filter band at: 1.8–6.1 × 10^3^ Hz (blue) band, 6.1–9.8 × 10^3^ Hz (red) and 9.8–12.8 × 10^3^ Hz (green); for: (**a**) 200 rpm, and (**b**) 500 rpm.

Testing whether the number of FTF harmonics is related to the number of damaged REs has also been performed. This was done by repeating the analysis with 5 damaged REs, only for an F4 grade fault and under three rotating regimes (200, 350 and 500 rpm). Figure 19 presents this analysis for 200 and 500 rpm, where the presence of certain FTF harmonics are observed; and depending upon the analysis band, the FTF harmonics are more representative. In this case, in the presence of 5 REs with faults the presence of the first 5 harmonics is not evident, ruling out the hypothesis assumed for the case of the 3 damaged REs, where a large presence of 3 FTF harmonics was observed.

## 4. Conclusions

A data acquisition unit has been developed, with a 16-bit resolution and high frequency data capture, reaching 110, 65, 45 and 35 kHz using 1, 2, 3 and 4 channels respectively. Although it is geared to the analysis of bearing vibrations, it is a multi-purpose unit that can measure any signal measured in volts, in the 0–5 V range. Its efficiency has been tested by means of a DoE, prepared for the analysis of faults in bearings. The data acquisition system suggested in this analysis has been tested to effectively meet the conditions required for vibration analysis, confirming that the development of a low-cost diagnostics unit with a high sampling frequency in each channel base on this prototype is feasible. Furthermore, as this unit is RPi based, data storage is carried out directly in its memory bypassing any connection to an external computer for data transmission, while enabling remote communication for remote record capture control. In addition, its design enables implementation in difficult access areas, due to its compact size.

However, the unit designed requires a signal resampling stage, due to a variation in the sampling frequency, a characteristic required for data analysis in the frequency domain. This drawback will be addressed in future developments, in order to ensure the stability of the recording frequency, and therefore reduce the total working time.

The data represented have been processed by means of envelope analysis, enabling the satisfactory identification of the faults generated in the rolling elements. Through which, the RE defects observed do not present a classical envelope vibration mode, but rather manifest themselves through impulsive content bands in their frequency (BSF) and harmonics. Another significant characteristic to point out, is the close relationship of the BSF with FTF, observing that as the fault severity grows the FTF frequency amplitude and harmonics increase. In addition, the influence of the vibration rate rotation has been proven, drastically increasing the frequency amplitude associated to the fault, as the speed increase. These results, consistent with other researchers [23,31], validate the efficiency of the recording unit designed.

The clear advantage of this unit in comparison to other commercial units, considered to be low-cost (described in Section 3.1), is the two-fold increase in recording capacity, plus its reduced cost at less than half the price. There are other low-cost commercial units with higher capabilities (myDAQ from National Instruments), with a maximum sampling frequency of 200 kHz; however, their price is five times more in comparison with the unit suggested.

As future processing work, other types of analyses will be implemented, such as spectral kurtosis to better identify the analysis band and the analysis of independent components in order to identify the signals of the bearing under analysis, in order to implement an intelligent fault detection system based on artificial intelligence (neural networks, support vector machines) using these outputs.

## Figures and Tables

**Table 1:** Fundamental frequencies of the 22205E1KC3 bearing at different regimes.

22205E1KC3	Frequency		Regime (rpm)		
			200	350	500
BPFO	6.1852	× f	20.62	36.08	51.54
BPFI	8.8148	× f	29.38	51.42	73.46
BSF	5.4030	× f	18.01	31.52	45.03
FTF	0.4123	× f	1.37	2.41	3.44

**Table 2:** Characteristic frequencies of the 6304-2R bearing at different regimes.

6304-2R	Frequency		Regime (rpm)		
			200	350	500
BPFO	2.5783	× f	8.59	15.04	21.49
BPFI	4.4398	× f	14.80	25.90	37.00
BSF	3.5241	× f	11.75	20.56	29.37
FTF	0.3687	× f	1.23	2.15	3.07

**Table 3:** Factors and levels considered in the design of experiment.

**Study Factor**		**Levels**					**Units**
		**F1**	**F2**	**F3**	**F4**	**F5**	
RE	Area	0	11.05	11.57	11.7	13	mm^2^
	Depth	0	0.006	0.014	0.019	0.027	mm
**Control Factor**		**Levels**					**Units**
		**R1**		**R2**	**R3**		
Regime		200		350	500		rpm

**Table 4:** Uncontrolled variables in the design of experiment.

Uncontrolled Factors	Value	Units
Ambient Temperature	20 to 24	°C
Operating temperature of casing	35 to 45	°C
Relative humidity	40 to 52	%
Atmospheric Pressure	1010 to 1016	hPa

**Table 5:** Details of the recording capacity of the equipment per channel.

No. of Channels	Sampling Frequency [kHz]	Standard Deviation [Hz]
1	110	16,280
2	65	7085
3	45	4403
4	35	3057

## Supplementary Materials

Database available at: https://zenodo.org/record/3898942#.Xuo94kUzZPY.
